# Dynamics and mechanisms of clonal expansion of HIV-1-infected cells in a humanized mouse model

**DOI:** 10.1038/s41598-017-07307-4

**Published:** 2017-07-31

**Authors:** Yorifumi Satou, Hiroo Katsuya, Asami Fukuda, Naoko Misawa, Jumpei Ito, Yoshikazu Uchiyama, Paola Miyazato, Saiful Islam, Ariberto Fassati, Anat Melamed, Charles R. M. Bangham, Yoshio Koyanagi, Kei Sato

**Affiliations:** 1 International Research Center for Medical Sciences (IRCMS), Kumamoto University, Kumamoto, 860-0811 Japan; 2 Center for AIDS Research, Kumamoto University, Kumamoto, 860-0811 Japan; 3 Priority Organization for Innovation and Excellence, Kumamoto University, Kumamoto, 860-0811 Japan; 4 Laboratory of Systems Virology, Institute for Frontier Life and Medical Sciences, Kyoto University, Kyoto, 606-8507 Japan; 5 Division of Human Genetics, Department of Integrated Genetics, National Institute of Genetics, Shizuoka, 411-8540 Japan; 6 Department of Medical Physics, Faculty of Life Sciences, Kumamoto University, Kumamoto, 862-0976 Japan; 7 Division of Infection and Immunity, University College London, London, WC1E 6BT United Kingdom; 8 Department of Immunology, Division of Infectious Diseases, Imperial College London, London, W2 1PG United Kingdom; 9 CREST, Japan Science and Technology Agency, Saitama, 322-0012 Japan

## Abstract

Combination anti-retroviral therapy (cART) has drastically improved the clinical outcome of HIV-1 infection. Nonetheless, despite effective cART, HIV-1 persists indefinitely in infected individuals. Clonal expansion of HIV-1-infected cells in peripheral blood has been reported recently. cART is effective in stopping the retroviral replication cycle, but not in inhibiting clonal expansion of the infected host cells. Thus, the proliferation of HIV-1-infected cells may play a role in viral persistence, but little is known about the kinetics of the generation, the tissue distribution or the underlying mechanism of clonal expansion *in vivo*. Here we analyzed the clonality of HIV-1-infected cells using high-throughput integration site analysis in a hematopoietic stem cell-transplanted humanized mouse model. Clonally expanded, HIV-1-infected cells were detectable at two weeks post infection, their abundance increased with time, and certain clones were present in multiple organs. Expansion of HIV-1-infected clones was significantly more frequent when the provirus was integrated near host genes in specific gene ontological classes, including cell activation and chromatin regulation. These results identify potential drivers of clonal expansion of HIV-1-infected cells *in vivo*.

## Introduction

Human immunodeficiency virus type 1 (HIV-1) is an exogenous retrovirus with worldwide distribution. HIV-1 infects CD4^+^ cells such as macrophages and helper T cells and causes acquired immunodeficiency syndrome (AIDS). Combination anti-retroviral therapy (cART) potently controls HIV-1 replication and prevents the onset of AIDS in most treated individuals^1^. However, cART does not eradicate the virus: a reservoir of HIV-1-infected cells persists despite prolonged therapy^2^, and infected individuals cannot interrupt treatment^3^. Furthermore, persistent HIV-1 infection causes various complications in infected individuals, such as HIV-associated neurocognitive disorders and a high incidence of certain cancers^4, 5^.

Since HIV-1 is a retrovirus, the viral RNA genome is reverse transcribed into a double stranded DNA. The viral DNA is integrated into the host genomic DNA to form a provirus, which is subsequently transcribed to generate new viral particles. In the absence of cART, there is active viral replication, resulting in a high viral load in the plasma. When infected individuals are treated with cART, the plasma viral RNA typically becomes undetectable by standard assays. In this situation, HIV-1 proviral DNA contributes to the maintenance of the viral reservoir, and hence much effort has been recently dedicated to study the distribution of HIV-1 proviral integration sites and the mechanisms of proviral reactivation^6–8^.

Previous studies have shown that HIV-1 is preferentially integrated into gene bodies with active transcription in *in vitro* infection^9, 10^. Recent studies of HIV-1 provirus from *ex vivo* samples of infected individuals have revealed several additional key observations. First, the strong selection pressure exerted on HIV-1 by escape from anti-retroviral drugs or anti-viral immunity, resulting in a high frequency of defective proviruses *in vivo* ^11–13^. Second, it has been reported recently that HIV-1-infected cells can undergo clonal expansion, as observed in infection with the related retrovirus human T-cell leukemia virus type 1 (HTLV-1)^12, 14, 15^. The clonal abundance of infected cells can be quantified by using integration site analysis^16, 17^. HIV-1 integration was frequently detected near cancer-related genes in the expanded clones of infected individuals^14, 15^. Clonal expansion may contribute to the maintenance of the HIV-1 reservoir^13, 18^, but the precise kinetics of generation of HIV-1-infected clones and the underlying mechanism of clonal expansion *in vivo* are still poorly understood.

*In vitro* studies have afforded much progress in HIV-1 research, including the development of anti-retroviral drugs. However persistent HIV-1 infection *in vivo* is associated with active host cell dynamics, i.e. differentiation, activation, quiescence, and homeostasis of CD4^+^ T cells, limiting the usefulness of *in vitro* cell culture systems^19^. Direct analysis of clinical samples is complicated by the wide variation between individuals in the time since initial infection, the genotype of the founder virus, and the immunological status of the host. To understand HIV-1 persistence in the host, *in vivo* models of infection are therefore required. Humanized mice transplanted with human CD34^+^ hematopoietic stem cells have been shown to be useful to study HIV-1 infection *in vivo* ^20–22^. One can study CD4^+^ T-cell dynamics in the humanized mouse model, including generation, differentiation, activation, and homeostasis^23^.

Here, we investigated whether the humanized mouse model recapitulates clonal expansion of HIV-1 infected cells as detected in patients, and whether it can provide mechanistic insight into the process of clonal selection of virus-infected cells. We found rapid generation of a large number of clones of HIV-1-infected cells and proliferation of individual clones within 2 weeks of infection. This period corresponds to the early stage of infection in human, before the emergence of the host immune response. Furthermore, in the expanded clones we found significant enrichment of HIV-1 proviruses integrated near genes involved in lymphocyte activation and chromatin regulation, providing the evidence for underlying mechanism of clonal expansion of HIV-1-infected cells.

## Results

### HIV-1 infection in the humanized mice: plasma viral load, CD4^+^ T-cell count and proviral load

**Figure 1:**
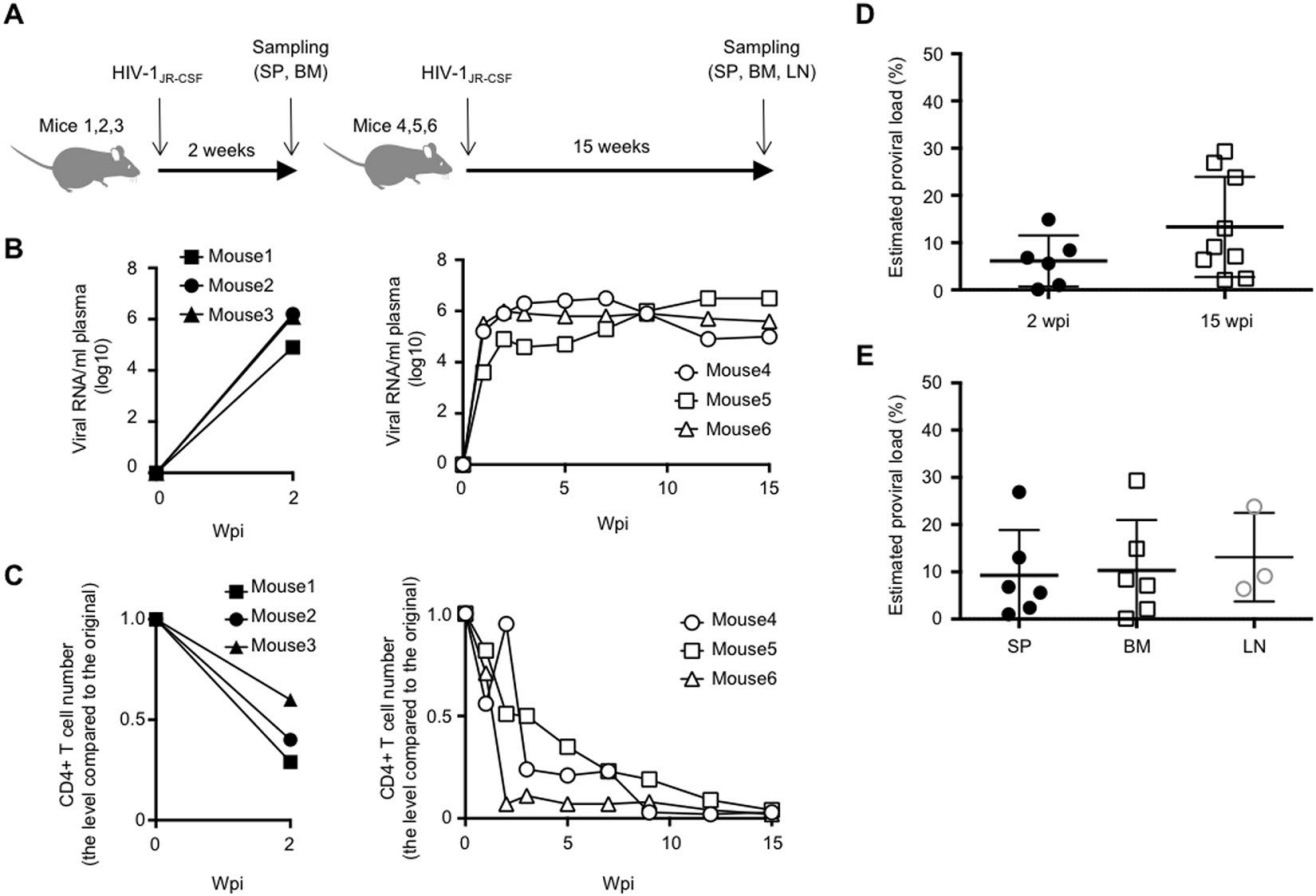
HIV-1 infection in the humanized mice. (**A**) Diagram showing the experimental design. Bone marrow (BM) and spleen (SP) were collected from 2 wpi mice and BM, SP, and lymph node (LN) were collected from 15 wpi mice for clonality analysis. Longitudinal analysis of plasma viral load (**B**) and CD4^+^ T-cell number (**C**) in 2 wpi and 15 wpi humanized mice. (**D**) Comparison of proviral load between 2 wpi and 15 wpi mice. (**E**) Comparison of proviral load among different tissues, SP, BM, and LN.

We transplanted human CD34^+^ hematopoietic stem cells into the liver of new-born recipient NOD/SCID/*Il2rg* ^null^ mice (NOG mice). After 10–12 weeks post-transplantation, the humanized mice (n = 6) were intraperitoneally infected with the CCR5-tropic HIV-1_JR-CSF_. We divided the six mice into two groups; respectively 2 weeks post infection (wpi) and 15 wpi (Fig. 1A and Supplementary Table S1). The plasma viral RNA was around 10^6^ copies/mL after 1–2 wpi and this high viremia was still present at 15 wpi (Fig. 1B). The CD4^+^ T-cell count fell to half the initial level by 2 wpi and to less than 5 percent of the initial level by 15 wpi (Fig. 1C), consistent with our previous report^24^. To analyze HIV-1 proviruses, we extracted genomic DNA from spleen (SP) and bone marrow (BM) of 2 wpi mice. We also extracted DNA from lymph node (LN) of 15 wpi mice as well as SP and BM. The estimated proviral load (PVL) was much higher than that typically observed in peripheral blood mononuclear cells (PBMCs) of infected individuals (Fig. 1D)^25^, and there was no significant difference in PVL between different tissues (Fig. 1E). We also observed wide variation in estimated PVL levels among individual mice and different tissues (Fig. 1D and E).

### Clonality of HIV-1 infected cells in infected humanized mice

**Figure 2:**
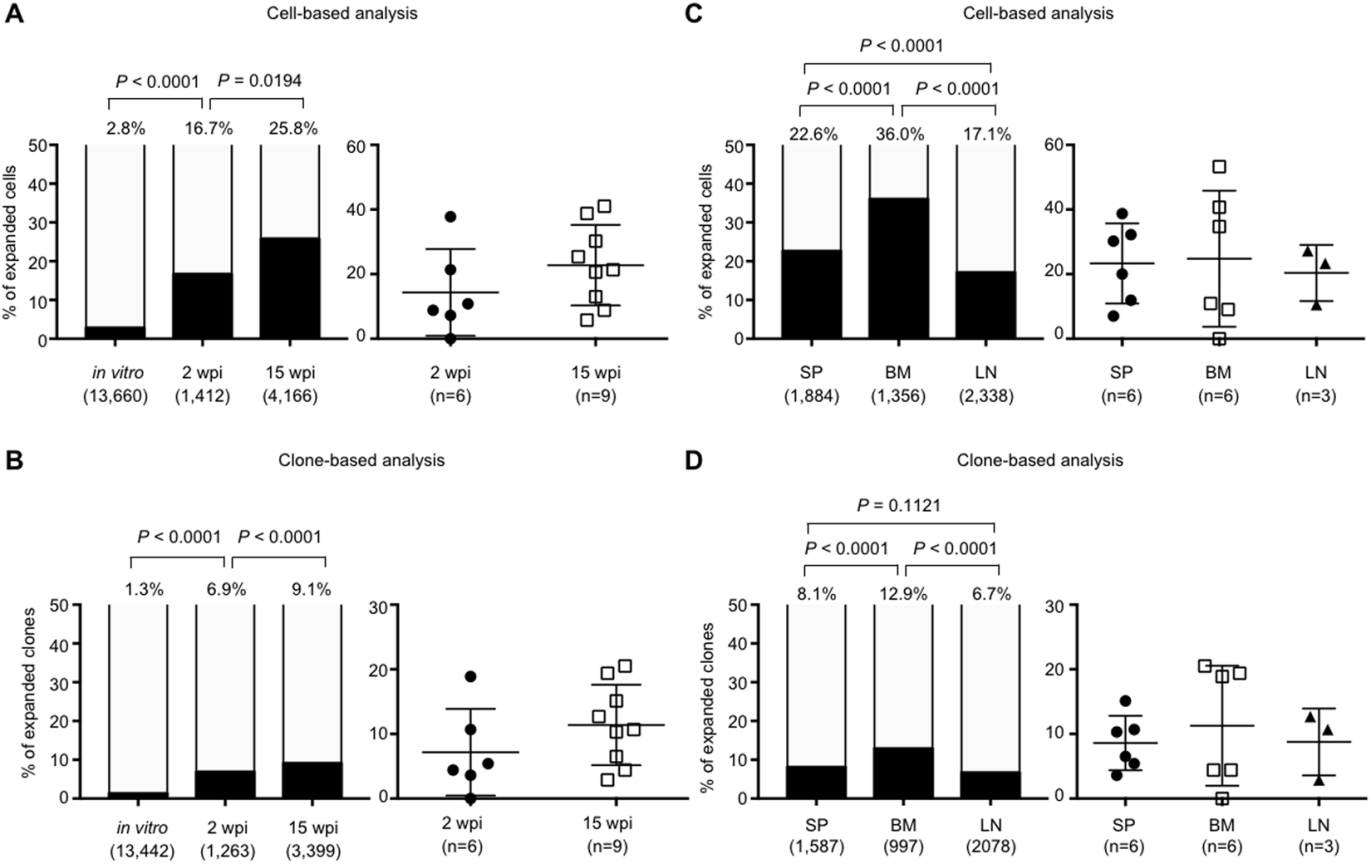
Cell-based and clone-based integration site analysis. (**A**) The percentages of expanded cells within the total number of infected cells were analyzed using genomic DNAs from tissues of 2 wpi or 15 wpi mice. DNA extracted from Jurkat cells infected with HIV-1 *in vitro* was analysed as a control. Cumulative data are shown in the left panel. The value for each individual mouse is shown in the right panel. (**B**) Clone-based analysis of 2 wpi or 15 wpi mice. (**C**) Comparison of the degree of clonal expansion at different tissues. Cumulative results of cell-based analysis are shown in the left panel, and the value of each individual mouse is shown in the right panel. (**D**) Clone-based analysis is also shown. The number of infected cells or clones in each sample is shown under the graph of the left panel. The number of mice or tissues analysed is shown under the graph of the right panel.

**Figure 3:**
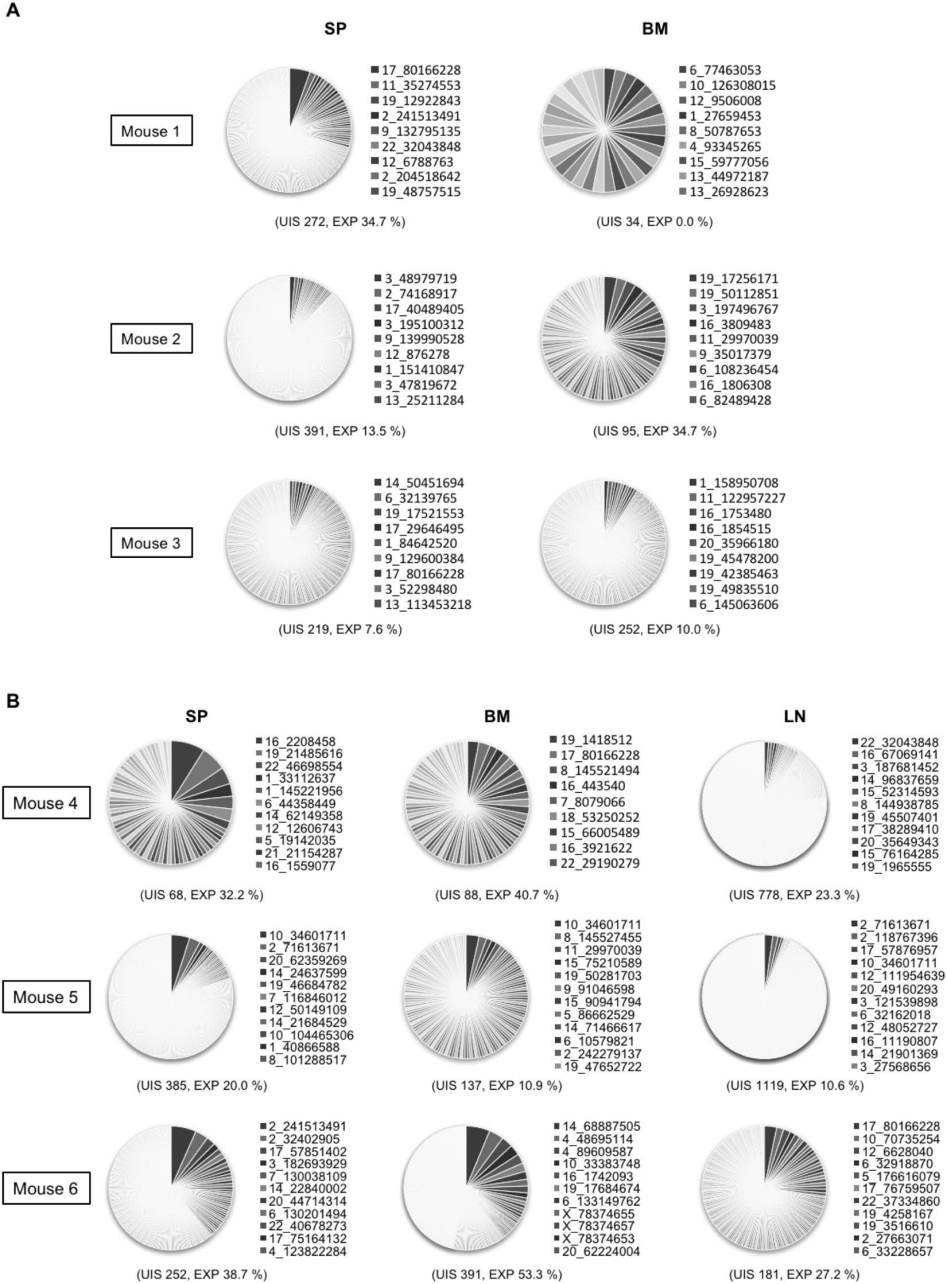
Degree of clonal expansion of HIV-1-infected cells in individual mice. (**A**,**B**) Each pie chart graph shows the relative abundance of each individual clone in 2 wpi (**A**) and 15 wpi (**B**) mice. The area represents the relative degree of expansion of each individual clone in the tissue sample. DNA samples were analysed according to the experimental procedure shown in Supplementary Figure S1 and described in Methods. UIS: Number of unique integration site, EXP: % of expanded clone. A list of integration sites of the highly-expanded clones is shown on the right side of the pie chart as chromosome, positions.

To investigate if HIV-1 infection resulted in clonal expansion of HIV-1-infected cells in the humanized mice, we mapped and quantified HIV-1 integration sites by using linker-mediated PCR as previously described^16^, with minor modifications (Supplementary Fig. S1). We also calculated clonal abundance using information on the DNA shear site^17^. In total, we identified 4,662 and 13,442 unique HIV-1 integration sites in humanized mice and in *in vitro* infection, respectively. Here, we defined an infected clone that was detected more than once in the same sample as an “expanded clone”, whereas an infected clone detected only once was called a “singleton clone”. We found that the extent of clonal expansion of the infected cells in 2 wpi mice was much greater than that of cells infected *in vitro* (*P* < 0.0001, Fig. 2A). In 2 wpi mice, 87 clones (236 cells) were expanded, whereas 1,176 clones (1,176 cells) were identified as singleton clones. In 15 wpi mice, we identified 309 expanded clones (1,076 expanded cells) and 3,090 singleton clones (3,090 single cells) (Fig. 2A and B). The proportion of expanded clones increased significantly from 2 wpi to 15 wpi, as judged by both cell-based and clone-based analysis (*P* = 0.0194 and *P* < 0.0001, respectively, Fig. 2A and B). Clonal expansion was most evident in BM, although there was wide variation between individual mice (Fig. 2C and D). The relative clonal abundance of each infected clone is shown in Fig. 3A and B. The expanded clones represented between 7.6% and 34.7% of total infected cells in 2 wpi mice (except for one BM tissue with very low PVL, 0.1%) and between 10.6% and 53.3% in 15 wpi mice. These results indicate that HIV-1 infected cells underwent clonal expansion within 2 weeks in the humanized mouse model.

### HIV-1 preferentially integrated into the gene body of highly-expressed host genes in the humanized mice

**Figure 4:**
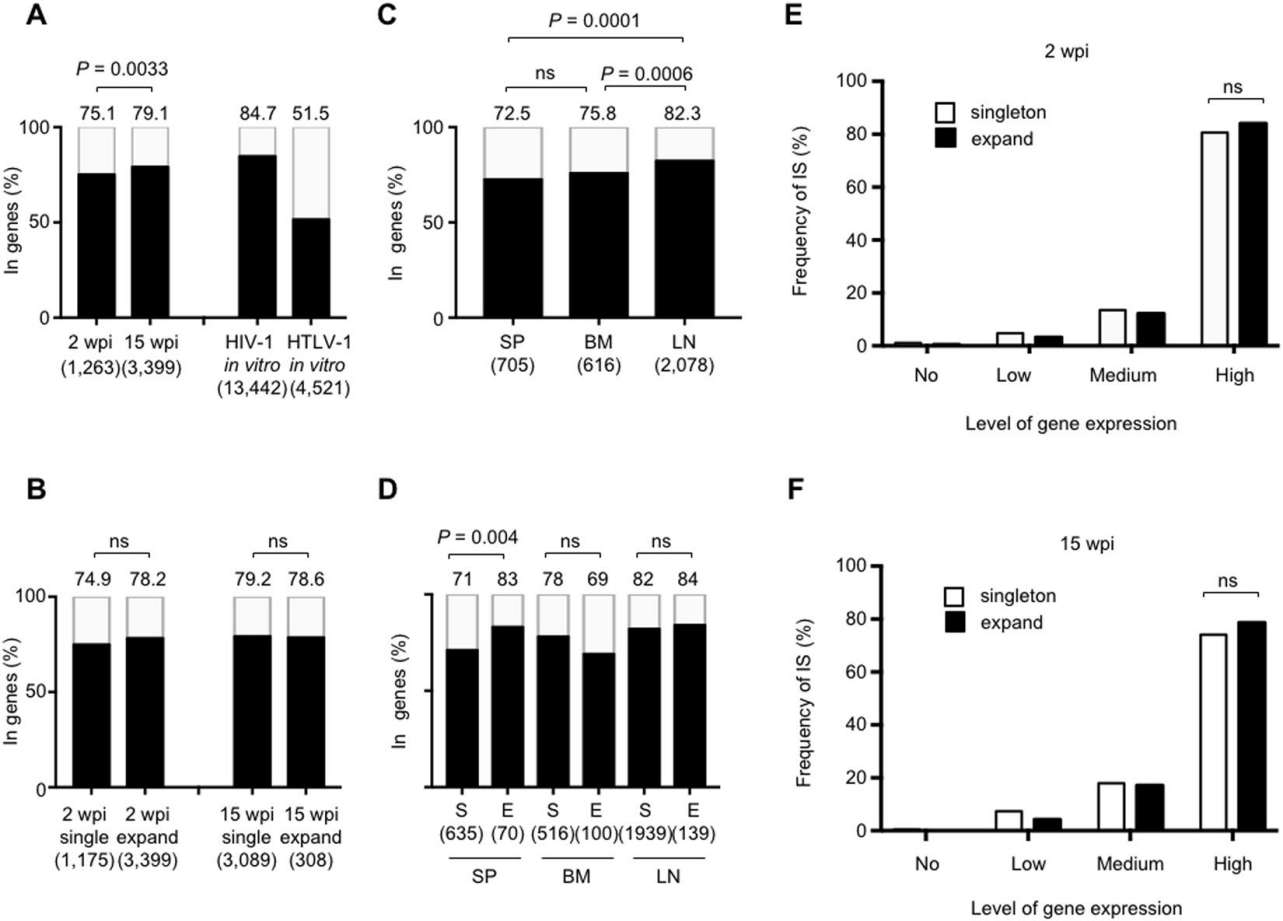
Relationship between HIV-1 integration sites and the host genes. (**A**) Frequencies of the integration sites located in genes in 2 wpi or 15 wpi mice are shown in the bar graph. Jurkat cells infected with HIV-1 or HTLV-1 *in vitro* were used as controls. (**B**) Comparison of the frequency of expanded and singleton clone in 2wpi and 15 wpi mice. (**C**) Frequencies of integration sites within genes are shown for different tissues. (**D**) Comparison of the frequency between expanded and singleton clones in different tissues. S: singleton clones, E: expanded clones. Numbers above the graph indicate the percentage. The number of infected cells or clones in each sample is shown under the graph. (**E**,**F**) Relationship between integration sites and level of expression of the corresponding host genes in 2 and 15 wpi mice. The cumulative results from different tissues of 2 wpi mice (**E**) and 15 wpi mice (**F**) are shown in the bar graphs. ns: no significant difference.

It has been reported that HIV-1 preferentially integrates into actively transcribed genes in human cells^9, 10^. We next analyzed the distribution of the HIV-1 integration sites (IS) in the humanized mice. Integration sites in cells infected *in vitro* with HIV-1 or human T-cell leukemia virus type 1 (HTLV-1) were analyzed as controls. Consistent with previous reports^9, 26^, HIV-1 preferentially integrated within host genes. The global distribution of HIV-1 integrations *in vitro* was similar to the distribution *in vivo*, whereas HTLV-1 integration *in vitro* did not show such a tendency (Supplementary Fig. S2). Closer analysis showed that HIV-1 IS in the humanized mice were frequently located inside the gene body (Fig. 4A). The frequency of integrations within genes was lower than that seen in *in vitro* HIV-1 infection (84.7%), but much higher than that in HTLV-1 infection *in vitro* (51.5%) (Fig. 4A). The frequency of integration within genes was higher in 15 wpi mice than in 2 wpi mice (79.1% and 75.1%, respectively), which reached statistical significance (*P* = 0.0033). There was no significant difference in the frequency of HIV-1 integration within genes between expanded and singleton clones, either in 2 wpi or 15 wpi mice (Fig. 4B). We also analyzed HIV-1 integration into exonic or intronic regions of host genes, and found a high frequency of HIV-1 integration into intronic regions, as reported previously^10, 12^ (Supplementary Fig. S3). There was a significantly increased frequency of HIV-1 integration into gene bodies in LN (82.3%) compared with SP (72.5%) or BM (75.8%) (Fig. 4C). In SP the frequency of integration into gene bodies was significantly higher in expanded clones than in singleton clones (*P* = 0.004, Fig. 4D), but not in BM and LN. There was no significant association between the transcriptional orientation of the HIV-1 provirus and that of the host gene, either in the whole proviral population (Supplementary Fig. S4A and B) or in any tissue examined (Supplementary Fig. S4C and D).

Previous reports have shown that HIV-1 is frequently integrated into host genes that are highly expressed^9, 12^. To investigate whether this is also the case in our model, we first performed RNA-sequencing (RNA-seq) analysis using CD4^+^ T cells from SP of two humanized mice without HIV-1_JR-CSF_ infection to generate average transcription values of each gene, and then compared the distribution of HIV-1 integration sites and the intensity of host gene expression. The results showed frequent integration of HIV-1 into highly expressed genes in both 2 wpi and 15 wpi mice (Fig. 4E and F), consistent with previous studies on PBMCs from HIV-1-infected individuals^12^. However, the frequency of integration into highly expressed genes did not differ significantly between singleton and expanded clones, either in 2 wpi or 15 wpi mice (Fig. 4E and F).

### Enrichment of HIV-1 integration sites in expanded clones near host genes associated with lymphocyte activation and chromatin regulation

**Figure 5:**
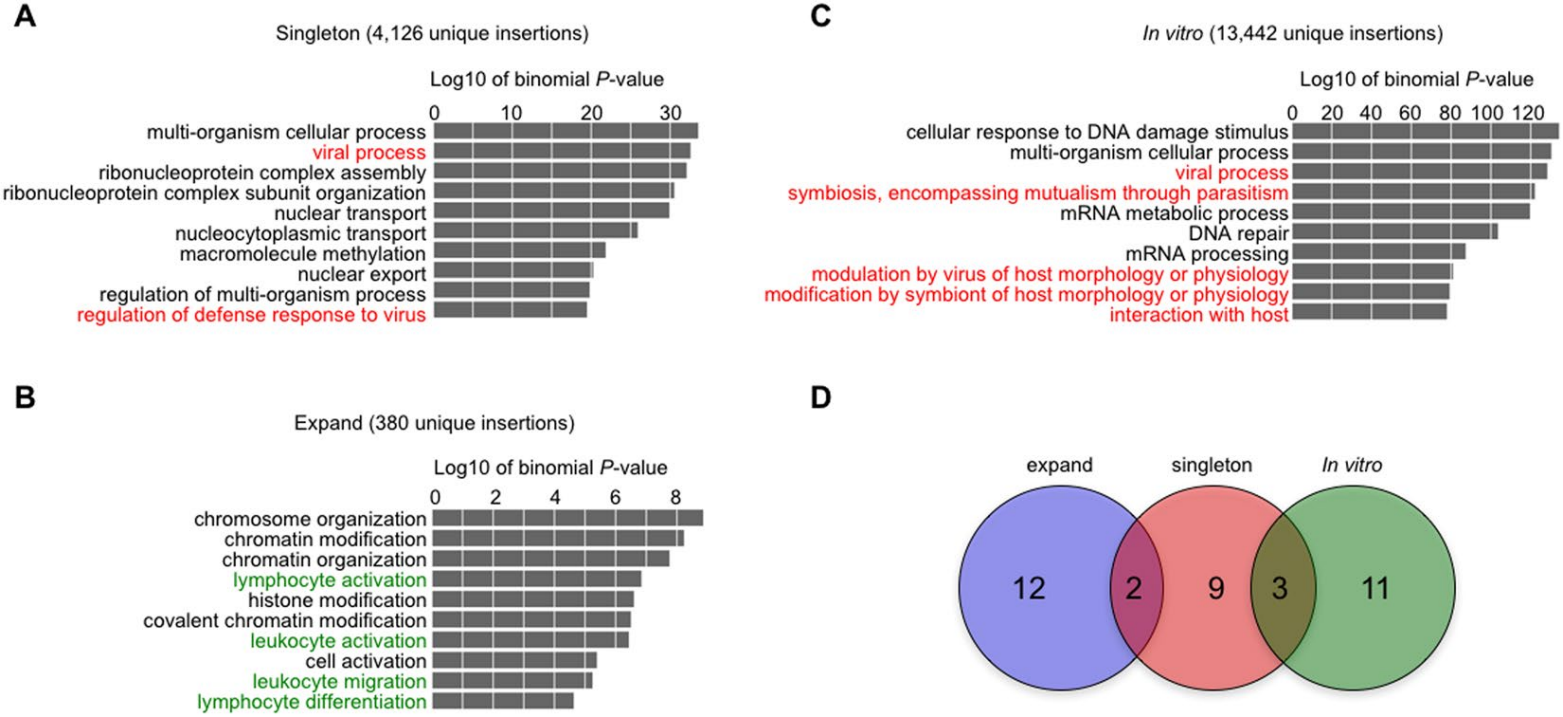
Gene ontology enrichment analysis of HIV-1 integration sites. (**A**,**B**) GO enrichment analysis of the HIV-1 integration sites in singleton (**A**) and expanded clones (**B**). (**C**) The GO enrichment analysis for *in vitro* integration sites is shown as a control. The numbers above the graphs represent – log10 of binomial *P*-value. There were 69, 14, and 105 GO terms enriched significantly in singleton clones, expanded ones, and *in vitro* infection, respectively. Term names shown in red and green are associated with “Host-pathogen interaction” and “leukocyte and lymphocyte activation”, respectively. (**D**) Venn diagrams showing the similarities and differences of enriched GO terms between each data set. For comparison, top 14 term names were selected from each data set.

In patients, HIV-1 IS in expanded clones were frequently detected near host genes associated with cell growth^12, 13^, but it is unclear whether this is a general mechanism. To address this issue, we performed gene ontology (GO) enrichment analysis on IS identified in this study. The analysis revealed that, while IS in singletons were enriched in genes associated with host-pathogen interactions (Fig. 5A and Supplementary Fig. S5), in expanded clones, IS were enriched into genes related to lymphocyte activation and chromatin regulation (Fig. 5B, Supplementary Fig. S6, and Supplementary Tables S2 and S3). The data from cells infected with HIV-1 *in vitro* showed a tendency similar to that observed in singleton clone (Fig. 5C). Comparisons between each enriched GO terms are shown in Fig. 5D. There were overlaps between singleton clones and *in vitro* infection; and between singleton and expanded clones, but not between *in vitro* infection and expanded clones when we analyzed the top 14 GO terms from each group. In expanded clones, HIV-1 IS were more frequently near genes related to lymphocyte activation and chromatin regulation than in singleton clones, which reached statistical significance (Table 1). These results indicated potential drivers of clonal expansion of HIV-1-infected cells *in vivo*.

**Table 1:** List of GO terms enriched in expanded clones.

GO_Id	GO_name	Odds ratio	*P*-value	*Q*-value
GO:0050900	leukocyte migration	0.522	0.008	0.059
GO:0016570	histone modification	0.568	0.009	0.059
GO:0051276	chromosome organization	0.664	0.009	0.059
GO:0016568	chromatin modification	0.635	0.009	0.059
GO:0016569	covalent chromatin modification	0.571	0.009	0.059
GO:0006325	chromatin organization	0.651	0.012	0.065
GO:0045321	leukocyte activation	0.643	0.019	0.079
GO:0046649	lymphocyte activation	0.627	0.018	0.079
GO:0001775	cell activation	0.702	0.040	0.148

GO terms included in the Venn diagrams (Fig. 5D) were analyzed to determine statistical significance. *P*-value and *Q*-value was determined by using Fisher’s exact test or Storey Tibshirani method^52^, respectively. Odd ratio was calculated as S_P_E_A_/S_A_E_P_ (S_P_; number of singleton IS present nearby genes related to a specific GO term, S_A_; number of singleton IS absent nearby those genes, E_P_; number of expanded IS present nearby those genes, and E_A_; number of expanded IS absent nearby those genes).

### Some expanded clones were detectable in multiple organs

**Figure 6:**
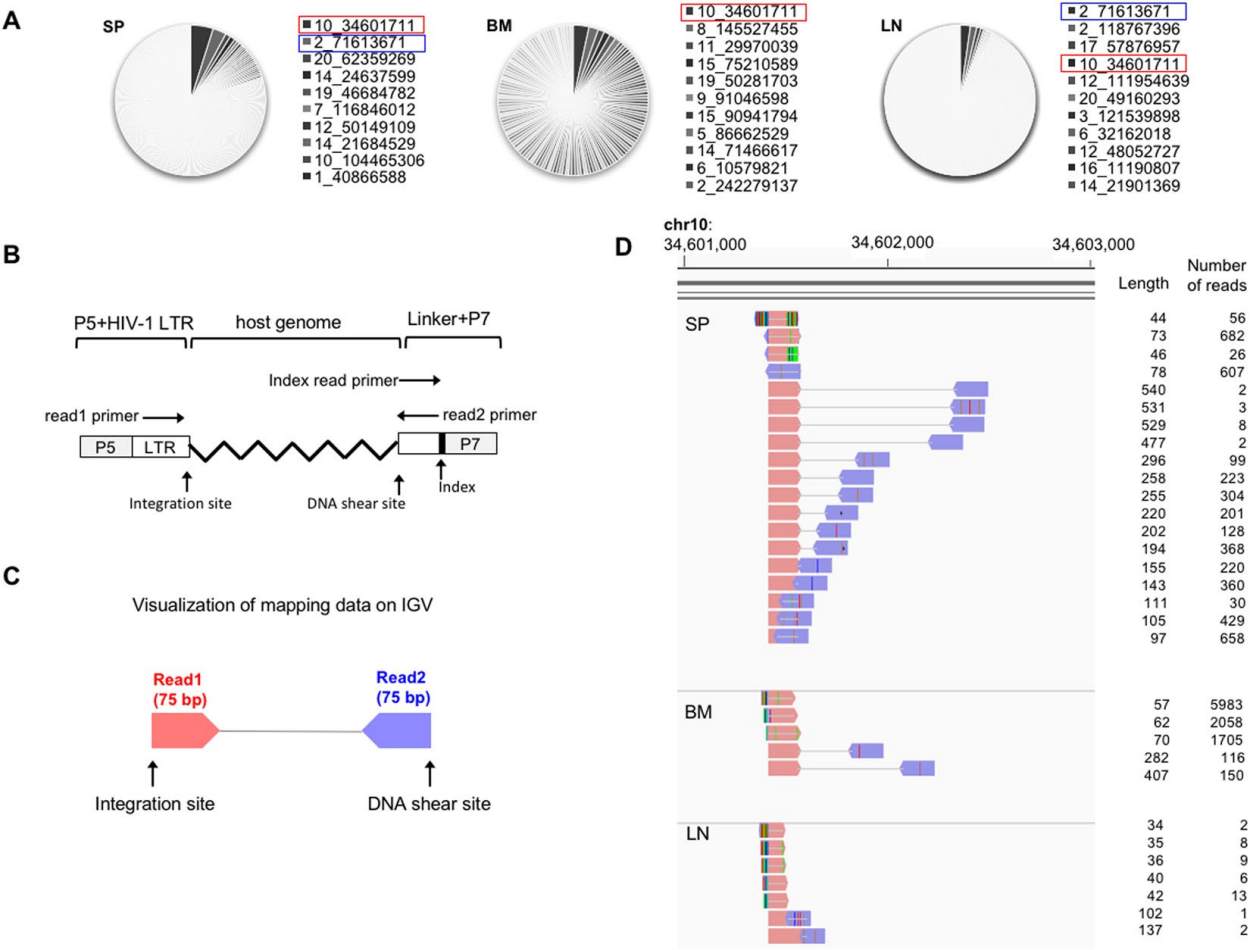
Evidence of systemic expansion of HIV-1-infected clones. (**A**) Pie charts of the abundance of HIV-1-infected clones in the mouse no. 5 shown in Fig. 3B. Red rectangles highlight a highly expanded clone detected in SP, BM, and LN. Blue rectangles highlight a highly expanded clone in SP and LN (Supplementary Fig. S7). (**B**) Schematic diagram to show the positions of each primer used for Illumina sequencing. (**C**) Schematic diagram for visualizing mapped data on IGV software. (**D**) Visualization of sequencing data of an expanded clone detected in all tissues analyzed. The length between the integration site and the DNA shear site is shown on the right side of each amplicon. The number of reads is also shown on the right side of each amplicon.

Almost all previous studies on the clonality of HIV-1 infected cells focused on CD4^+^ T cells or mononuclear cells in peripheral blood, except for one case report^18^. In order to analyze whether the expansion of HIV-1-infected cells in the humanized mouse is systemic or a local phenomenon, we compared the data on integration sites among different organs in a 15 wpi mice (mouse no. 5, Fig. 6A). Because of the high sensitivity of the high-throughput sequencing and analysis pipeline, there is a risk of technical false positives, due to an overflow of highly abundant sequence reads into other samples (Supplementary Fig. S1), which could lead to an overestimation of the extent of systemic expansion of HIV-1-infected cells. To minimize the risk of such false positive results, we used strict criteria to evaluate clonal expansion. We analyzed multiple samples in the same run and then separated the data by the sequence of index reads (Supplementary Fig. S1). The multiplexing step can also generate contamination of sequence reads across samples. To reduce the possibility of errors caused by the multiplexing, we filtered the data based on the quality of the index reads. We utilized 8-bp index reads and selected high-quality reads (Phred quality score > 30) only. In theory, the risk of misidentification of an index was less than 1 × 10^−15^. In addition, we discarded sequence reads that contained more than one low quality residue (Phred quality score < 20) in the Read 1 and Read 2 data, to reduce the possibility of false positives due to sequencing or mapping errors. These strict criteria enabled us to minimize the chance of overestimation of the frequency of abundant clones. The results from a 15-wpi mouse showed that several expanded clones were detectable in multiple tissues (Fig. 6A). For example, an HIV-1-infected expanded clone with the integration site in chr10: 34,601,711 was detected in SP, BM and LN. In each such clone, the integration site was identical, but the DNA shear site was different among those tissues, suggesting that the detection of the same clone in different tissue was not the result of cross-contamination but rather represented true systemic expansion of the HIV-1 infected clone (Fig. 6B and D). Using the same strict criteria, other expanded clones were detected in both SP and LN (Supplementary Fig. S7) and also in other 15 wpi mice (Supplementary Fig. S8).

## Discussion

The high-throughput sequencing of randomly-sheared DNA has made it possible to comprehensively quantify the clonality of retrovirus-infected cells^16, 17^, and the approach has led to recent reports of clonal expansion of HIV-1–infected cells in the peripheral blood of infected individuals^12, 14, 15^. These important findings raise the question whether clonally expanded cells contribute to the latent viral reservoir. For instance, a previous report suggested that clonally expanded HIV-1-infected cells might not contribute to maintain the viral reservoir because all expanded clones appeared to contain defective HIV-1 proviruses^12^. In contrast, another study showed that expanded clones that accumulate near cancer tissue in HIV-1-infected patients have the capacity to produce infectious viral particles^18^. A recent comprehensive study by using PBMCs and LNs in individuals with natural virologic control demonstrated that some expanded clones could induce virion production after T-cell stimulation^13^. These data suggest that at least some expanded HIV-1 infected clones can function as a viral reservoir.

Several critical issues remain to be addressed regarding clonally expanded HIV-1-infected cells, such as the kinetics of their emergence and their tissue distribution. The findings reported in this study suggest that clonal expansion of HIV-1 infected cells is rapid and happens within two weeks of infection. Furthermore, such expanded clones are distributed throughout the lymphoid organs in our humanized mouse model. Their survival and expansion is remarkable in the face of a steep drop in the total number of CD4^+^ T cells in 15 wpi mice (Fig. 1C), suggesting a strong selective advantage. Since the population size and type of CD4^+^ T-cell subsets at 15 wpi can be changed compared to those at 2 wpi, it is quite plausible that the estimates of infected cells types (clonal and singleton) can be impacted by these effects. This possibility will be addressed in future investigations. Also, it will be interesting to investigate if some of these expanded clones bear a functional, yet latent provirus and if they belong to specific classes of memory CD4^+^ T cells^27, 28^.

Another critical question that remains to be addressed is the mechanism that causes clonal expansion of HIV-1 infected cells. Previous studies demonstrated frequent integration into cell growth and cancer-related genes in expanded clones^14, 15, 29^, thus HIV-1 integration might induce aberrant expression of cancer-related genes, resulting in enhanced cell proliferation. Alternatively, some HIV-1 infected cells might have a higher proliferative capacity, independently of any effect of the integrated provirus. In this study, we observed frequent HIV-1 IS into genes associated with lymphocyte activation, proliferation, and chromatin regulation in expanded clones, but less so in singletons. Activated, proliferating CD4^+^ T cells are characterized by a high expression of the genes related with cell activation. Because HIV-1 preferentially integrates into highly transcribed genes^9, 12^, a scenario is plausible whereby a significant number of HIV-1 infections occurs in actively proliferating CD4^+^ T cells, resulting in frequent proviral integration near or within genes associated with cell activation. These rapidly proliferating CD4^+^ T cells likely give rise to expanded clones, some of which may be long-lived if the provirus is defective or later becomes latent, for example by transcriptionally interference^30, 31^. This picture is consistent with the observation that HIV-1 persists in memory T-cells, which undergo mitosis more frequently than naïve T cells^13, 32^.

As we have shown in this study, the humanized mouse model should be useful to address key questions on clonal expansion of HIV-1 infected cells for the following reasons:

First, all tissues are accessible in the mice, in addition to PBMCs. Most of previous studies were limited to PBMCs yet HIV-1 infection involves tissues such as the spleen, lymph nodes, the gastrointestinal mucosa and the central nervous system^33^. We show here that the clonal expansion of HIV-1-infected cells is not a local phenomenon but systemic because we observed the same expanded infected clone in multiple organs (Fig. 6). We found that clonal expansion was more evident in BM than in SP or LN (Fig. 2C and D). The frequency of integration into host genes was significantly higher in LN than other tissues (Fig. 4C) and it was close to that in cells infected *in vitro* (Fig. 4A), suggesting that *de novo* infection of HIV-1 in LN is more evident than other tissues. This idea is in line with findings in rhesus macaque infected with simian immunodeficiency virus and HIV-1 infected individuals with natural virologic control, where there is persistent viral replication in both follicular and non-follicular helper T cells in lymph nodes^13, 34^. Further studies are needed to understand the organ-specific dynamics of clonal expansion of HIV-1 infected cells.

Second, there is less heterogeneity in the humanized mice than in infected individuals. Previous studies identified thousands of integration sites in human subjects, but the number of patients analyzed was still small. Differences in terms of founder virus sequence, route of initial infection, duration of infection, treatment regimen and duration are likely to affect the clonal expansion of HIV-1 infected cells and their tissue distribution. To identify the fundamental underlying mechanisms of clonal expansion of infected cells, *in vivo* models with less heterogeneity should be advantageous. In this study, we demonstrated that clonal expansion occurred within 2 weeks of infection (Fig. 2A). In line with a previous report in human subjects^12^, the degree of clonal expansion of infected cells in the humanized mice increased with the duration of infection from 2 wpi (16.7%) to 15 wpi (25.8%) (Fig. 2A). In the mouse model, we will be able to analyze the kinetics of clonal expansion with greater confidence, because we will know the precise time of infection.

Third, the spread of HIV-1 in the humanized mouse occurs in the situation that the host immune response against virus is incompetent: the results may therefore mimic the spread of HIV-1 in early infection in the human, in whom an effective immune response to the virus is typically mounted after 1 to 4 months^35^. Because of the relatively short period of infection and lower selection pressure exerted by the host immune system, sequence variation of HIV-1 in the humanized mice should be much lower than that in human subjects^36^, reducing the frequency of undetected clones. Due to the design of the oligonucleotide primers used to analyze the integration sites, sequence variation in the primer-binding region of the provirus may preclude detection of certain clones. The fact that we may miss some clones in the integration site analysis must be taken into consideration when interpreting the clonality data. Several alternative methods for IS analysis to overcome this point are being developed^37, 38^.

In summary, we have shown rapid generation of a large number of clones of HIV-1-infected cells and expansion of individual clones in the humanized mouse. Individual expanded clones have disseminated into multiple organs at 15 weeks post infection. The HIV-1 provirus in expanded clones was integrated significantly more often near host genes with specific gene ontology such as lymphocyte activation and chromatin regulation, compared with the singleton clones. Although it still remains unknown whether HIV-1 integration directly causes clonal expansion of the infected host cells, our analyses of 18,104 integration sites from different data sets both *in vitro* and *in vivo* provide concrete evidence for the association between HIV-1 IS and the clonal expansion of HIV-1-infected cells and strengthened the application of the humanized mouse system to elucidate further mechanistic insights into persistent HIV-1 infection *in vivo*.

## Methods

### Ethics statement

All procedures including animal studies were conducted following the guidelines for the Care and Use of Laboratory Animals of the Ministry of Education, Culture, Sports, Science and Technology, Japan. The authors received approval from the Institutional Animal Care and Use Committees (IACUC)/ethics committee of Kyoto University institutional review board (protocol number D13–25). All protocols involving human subjects were reviewed and approved by the Kyoto University institutional review board. The study was carried out in accordance with the guidelines proposed in the Declaration of Helsinki. Informed written consent from human subjects was obtained in this study.

### HIV-1 infection to humanized mice

NOD/SCID/*Il2rg* ^null^ mice (NOG mice) were obtained from the Central Institute for Experimental Animals (Kawasaki, Japan)^39^. Human CD34^+^ hematopoietic stem cells were isolated from human fetal liver kindly provided by Dr. Dong Sung An (University of Los Angeles, USA). We generated humanized mice (NOG-hCD34 mice) as described previously^40, 41^. HIV-1_JR-CSF_ viral solution was prepared as previously described^42^. Briefly, pJR-CSF, an infectious molecular clone of HIV-1_JR-CSF_^43, 44^, was transfected into 293T cells. At 48 h post-transfection, the culture supernatant was harvested, centrifuged, and then filtered through a 0.45-μm-pore-size filter to produce virus solutions. Viral solution (1,500 infectious units [IU]) was intraperitoneally injected into the NOG-hCD34 mice. All mice were maintained in accordance with institutional guidelines for animal welfare at Kyoto University. Peripheral blood and plasma samples were collected at 0, 1, 2, 3, 5, 7, 9, 12, and 15 wpi as previously described^23, 36, 45, 46^. The amount of HIV-1 RNA in 50μL plasma was quantified by Bio Medical Laboratories, Inc. (Tokyo, Japan). CD4^+^ T-cell numbers in peripheral blood were calculated by flowcytometry using anti-CD4 antibody (RPA-T4; Biolegend). The mice were sacrificed at 2 or 15 wpi under anesthesia, and then human mononuclear cells (hMNCs) of the LN, SP, and BM of humanized mice were isolated and purified as previously described^23, 36, 41, 45, 46^. LNs were gently homogenized using a homogenizer pestle. SPs were crushed and rubbed on a steel mesh with 1-mm grids to generate single cell suspensions in RPMI1640 supplemented with 4% FCS. To collect BM, femurs were dissected at both ends and the interior was flushed with RPMI 640 supplemented with 4% FCS. hMNCs in the spleen and bone marrow of humanized mice were further purified from the MNCs of these organs using Ficoll-Paque (Pharmacia). The human MNCs were stored in Cell Banker (Juji Field Inc., Tokyo, Japan) at −80 °C until use.

### Preparation of HIV-1 or HTLV-1-infected cells *in vitro*

To prepare HIV-1-infected cells *in vitro* Jurkat E6.1 (ECACC) cells were infected cells with recombinant HIV-1 *in vitro* as described previously^47^. Briefly, Jurkat cells (30 mL at 0.8 × 10^6^/ml) were infected with VSV-G pseudotyped LAIΔenv at an MOI of 0.2. An aliquot of cells was analysed by FACS 36 hours post-infection and DNA was extracted from the remaining cells using Qiagen DNeasy Blood & Tissue kit following the manufacturer’s instructions. We also prepared HTLV-1 infected cells *in vitro* as describe before^16^. Briefly, Jurkat E6.1 (ECACC) cells were infected with HTLV-1 by co-cultivation with MT-2, a virus-producing cell line. To reduce contamination with MT-2 cells, we treated MT-2 with γ-irradiation before co-cultivation. DNAs were extracted from Jurkat cells infected with HTLV-1 using DNeasy Blood and Tissue kit (Qiagen).

### Linker-mediated (LM)-PCR

Integration site analysis of HIV-1 was performed using linker-mediated PCR and high-throughput sequencing as described previously with minor modifications^16^, which is the same strategy as a previous report analyzing PBMCs of HIV-1-infected individuals^14^. Briefly, 1 microgram genomic DNA was fragmented by sonication with a Covaris S220 (Covaris, Inc., MA) or Picoruptor (Diagenode, S.A., Belgium) device to produce fragments in the range of 300–500bp. The DNA ends were repaired and a DNA linker was added. The junction between the 3′LTR of HIV-1 and the host genomic DNA was amplified with a primer targeting the 3′LTR and another targeting the linker. After a nested PCR, PCR amplicons were quantified using Illumina P5 and P7 primers. A list of oligonucleotides used in the LM-PCR is given in Supplementary Table S4. DNA libraries were sequenced by Illumina MiSeq as paired-end reads, and analysis performed on the resulting fastq files.

### Proviral load (PVL) measurement

We estimated the number of infected cells by quantifying the copy number of the *gag* gene, normalized to the copy number of the *ALB* gene, according to a previous report, with minor modification^25^. The presence of unintegrated 2-LTR circles^48^ in infected cells can contribute to the copy number of *gag* in the assay. Therefore we subtracted the copy number of the 2-LTR DNA from the total copy number of *gag*. The proviral load was calculated as follows: PVL (%) = ((copy number of gag) – (copy number of 2-LTR))/((copy number of albumin)/2) × 100. Primer sequences are listed in Supplementary Table S5. In this PVL measurement, proviruses defective of *gag* region and the presence of unintegrated 1-LTR circles could affect the PVL value. Thus, we used “estimated PVL” as the y-axis label of proviral load measurement (Fig. 1C and D).

### RNA-seq

To obtain the human CD4^+^ T cell fraction (CD45^+^CD3^+^CD8^−^) from the humanized mice, human splenic MNCs were stained with PE-conjugated anti-CD45-PE (HI30; Biolegend), APC-Cy7-conjugated anti-CD3 (HIT3a; Biolegend), and PE-Cy7-conjugated anti-CD8 antibody (HIT8a; Biolegend), and CD45^+^CD3^+^CD8^−^ cells were sorted with FACSJazz (BD Biosciences). The purity was >99%. RNA was extracted from CD4^+^ splenocytes using RNeasy (Qiagen). cDNA libraries were generated using TrueSeq RNA Access Library Prep Kit (Illumina). Sequencing was performed using TruSeq SR Cluster Kit v3 and TruSeq SBS Kit v3 – HS (50 cycle) (Illumina) by Medical Biological Laboratories Co., Ltd (Nagoya, Japan). HiSeq. 2000 (Illumina) generated fastq files with around 40–80 million reads per sample. RNA-seq data analysis was performed using CLC Genomics workbench 7.5 (CLC bio). We generated an average value of RPKM (Reads per kilobase of exon model per million mapped reads) for each human gene, and used these values to classify genes into 4 groups (no, low, medium, or high expression).

### High-throughput sequencing data analysis

Each cluster on the Illumina flow cell generated 3 fastq files, including Read1, Read2, and Index Read. Read1 corresponded to sequencing data generated by a primer within HIV-1 LTR (Fig. 6B) and Read2 to data generated by a primer within the linker. The Index Read corresponded to the 8-bp index sequence in the linker. First, we identified clusters on the flow cell with high sequencing quality of the Index Read (Phred quality score >30 at each position of 8-bp index read) using an R program kindly provided by Michi Miura (Imperial College London, UK). We next removed the linker sequence from Read1 and Read2, and then we selected reads with LTR sequence in Read1 (TGACA for HIV-1). After trimming the 5-bp viral sequence from Read1, we performed a further sequencing quality check using a bespoke Perl script (Amelieff, Tokyo, Japan). This software processes sequence data as follows: i) removes reads containing more than 80% bases with Q score less than 20, ii) removes reads less than 20 bp in length, and iii) removes unpaired reads. The cleaned sequencing reads were mapped to the HIV-1 genomes, (Genbank, K03455) with or without the human reference genome (hg19) using the BWA-MEM algorithm^49^. We used Samtools for further data processing, such as removal of multiple mapping to the reference genome^50^. Since the LTR sequence is identical, the mapping data contained information of both 5′- and 3′-LTR. To identify integration sites, we removed data from the 5′LTR. For the visualization of the mapped results as shown in Fig. 6C, we used Integrative Genomics Viewer (IGV) (http://software.broadinstitute.org/software/igv/). For clonality analysis of HIV-1-infected cells, we exported the files containing the information on integration sites, DNA shear sites, and number of reads (Fig. 6B). We then calculated the number of copies of each individual clone by counting the number of different shear sites present for each integration site^17^. There are two possibilities when we find two cells with the same IS. One possibility is that we are detecting the clonal expansion of one original clone. The other is HIV-1 integrating into the same integration site of two different host cells. The former possibility is far more likely than the latter. Because the human genome contains about 3 billion base pairs, the possibility of finding the same integration site by chance in two different clones is 0.00000003%. In the current study, we identified 4,662 unique integration sites in humanized mice. Therefore, the frequency that two different infected clones happen to have the same integration site is far less than 0.001%. Furthermore, the result shown in Fig. 2A, in which there were far less expanded clones *in vitro* infection than *in vivo*, strongly supports the former possibility. Thus, when we observed two cells with same integration site, we defined them as expanded clones in this study.

### Bioinformatic analysis

We generated a bed file from the exported file containing the information on integration sites. RefSeq gene data was obtained from UCSC tables (https://genome.ucsc.edu/). Positions of RefSeq genes were compared to the IS using the R package hiAnnotator (http://github.com/malnirav/hiAnnotator). We also performed Gene ontology (GO) analysis using GREAT, an online software application for gene annotations^51^ (http://bejerano.stanford.edu/great/public/html/index.php).

### Statistical analysis

Data were analyzed using a chi-squared test with Prism 7 software (GraphPad Software, Inc., CA) unless otherwise described. Statistical significance was defined as *P* < 0.05.

### Data availability

Fastq files obtained in this study have been deposited in the DNA Data Bank of Japan (DDBJ). (accession no. DRA005133). Data on the findings reported here are available from the corresponding author upon request.

## Electronic supplementary material

Supplementary information
