# Author Correction: Dynamics and mechanisms of clonal expansion of HIV-1-infected cells in a humanized mouse model

**DOI:** 10.1038/s41598-018-24607-5

**Published:** 2018-04-25

**Authors:** Yorifumi Satou, Hiroo Katsuya, Asami Fukuda, Naoko Misawa, Jumpei Ito, Yoshikazu Uchiyama, Paola Miyazato, Saiful Islam, Ariberto Fassati, Anat Melamed, Charles R. M. Bangham, Yoshio Koyanagi, Kei Sato

**Affiliations:** 1 International Research Center for Medical Sciences (IRCMS), Kumamoto University, Kumamoto, 860-0811 Japan; 2 Center for AIDS Research, Kumamoto University, Kumamoto, 860-0811 Japan; 3 Priority Organization for Innovation and Excellence, Kumamoto University, Kumamoto, 860-0811 Japan; 4 Laboratory of Systems Virology, Institute for Frontier Life and Medical Sciences, Kyoto University, Kyoto, 606-8507 Japan; 5 Division of Human Genetics, Department of Integrated Genetics, National Institute of Genetics, Shizuoka, 411-8540 Japan; 6 Department of Medical Physics, Faculty of Life Sciences, Kumamoto University, Kumamoto, 862-0976 Japan; 7 Division of Infection and Immunity, University College London, London, WC1E 6BT United Kingdom; 8 Department of Immunology, Division of Infectious Diseases, Imperial College London, London, W2 1PG United Kingdom; 9 CREST, Japan Science and Technology Agency, Saitama, 322-0012 Japan

Correction to: *Scientific Reports* 10.1038/s41598-017-07307-4, published online 31 July 2017

The Acknowledgements section in this Article is incomplete.

“We thank Michiyo Tokunaga for technical and secretary support, Alex Zhyvoloup for technical support, and Michi Miura for R program to perform quality check of Index reads. We also thank Heather Niederer for providing materials and Laurence Game and Marian Dore (Genomics Laboratory, Medical Research Council Clinical Sciences Centre, Hammersmith Hospital, London, UK) for sequencing of IS obtained from cells infected with HIV-1 or HTLV-1 *in vitro*. This study was supported by grants from the JSPS KAKENHI (26461428, 17K09016 and 15H04870 to Y.S., 16KK0206 to H.K., and 15K07166 to K.S.), AIDS International Collaborative Research Grant from the Ministry of Education, Science, Sports, and Culture, and Kumamoto University to Y.S., AMED (15Afk0410013h0001) to Y.K., CREST, JST to K.S., EU HIVINNOV (305137) to A. Fassati, and the Wellcome Trust (WT100291MA) to C.R.M.B. The funder has no role in the study design of the study, collection of data, its interpretation, or the discussion to submit the work for publication.”

should read:

“We thank Michiyo Tokunaga for technical and secretary support, Alex Zhyvoloup for technical support, and Michi Miura for R program to perform quality check of Index reads. We also thank Heather Niederer for providing materials and Laurence Game and Marian Dore (Genomics Laboratory, Medical Research Council Clinical Sciences Centre, Hammersmith Hospital, London, UK) for sequencing of IS obtained from cells infected with HIV-1 or HTLV-1 *in vitro*. This study was supported by grants from the JSPS KAKENHI (26461428, 17K09016 and 15H04870 to Y.S., 16KK0206 to H.K., and 15K07166 to K.S.), AIDS International Collaborative Research Grant from the Ministry of Education, Science, Sports, and Culture, and Kumamoto University to Y.S., AMED (15Afk0410013h0001) to Y.K., CREST, JST to K.S., EU HIVINNOV (305137) to A. Fassati, and the Wellcome Trust (WT100291MA) to C.R.M.B. The funder has no role in the study design of the study, collection of data, its interpretation, or the discussion to submit the work for publication. We would like to thank the UCLA CFAR Gene and Cellular Therapy Core Facility for providing human CD34+ hematopoietic stem cells (UCLA Center for AIDS Research NIH/NIAID 5P30 AI028697).”

