# Supplementary material for: Dynamics and mechanisms of clonal expansion of HIV-1-infected cells in a humanized mouse model
Source: Sci Rep. 2017 Jul 31;7:6913. doi: 10.1038/s41598-017-07307-4 (PMC5537293; doi:10.1038/s41598-017-07307-4)
Supplement: Supplementary file 1 — Supplementary information [file 41598_2017_7307_MOESM1_ESM.pdf]

Supplementary information:

Dynamics and mechanisms of clonal expansion of HIV-1-infected cells in a humanized mouse model

Yorifumi Satou[1,2,3†*], Hiroo Katsuya[1,2†], Asami Fukuda[1,3], Naoko Misawa[4], Jumpei Ito[5], Yoshikazu Uchiyama[6], Paola Miyazato[1,2], Mohammad S Islam[1,2], Ariberto Fassati[7], Anat Melamed[8], Charles R. M. Bangham[8], Yoshio Koyanagi[4], Kei Sato[4,9]

[1]International Research Center for Medical Sciences (IRCMS), [2]Center for AIDS Research, [3]Priority Organization for Innovation and Excellence, Kumamoto University, Kumamoto 860-0811, Japan, [4]Laboratory of Systems Virology, Institute for Frontier Life and Medical Sciences, Kyoto University, Kyoto 606-8507, Japan, [5]Division of Human Genetics, Department of Integrated Genetics, National Institute of Genetics, Shizuoka 411-8540, Japan, [6]Department of Medical Physics, Faculty of Life Sciences, Kumamoto University, Kumamoto 862-0976, Japan, [7]Division of Infection and Immunity, University College London, London WC1E 6BT, United Kingdom, [8]Department of Immunology, Division of Infectious Diseases, Imperial College London, London W2 1PG, United Kingdom, [9]CREST, Japan Science and Technology Agency, Saitama 322-0012, Japan

* Correspondence and requests for materials should be addressed to Y.S. (email: y-satou@kumamoto-u.ac.jp)

†These authors contributed equally to this work.

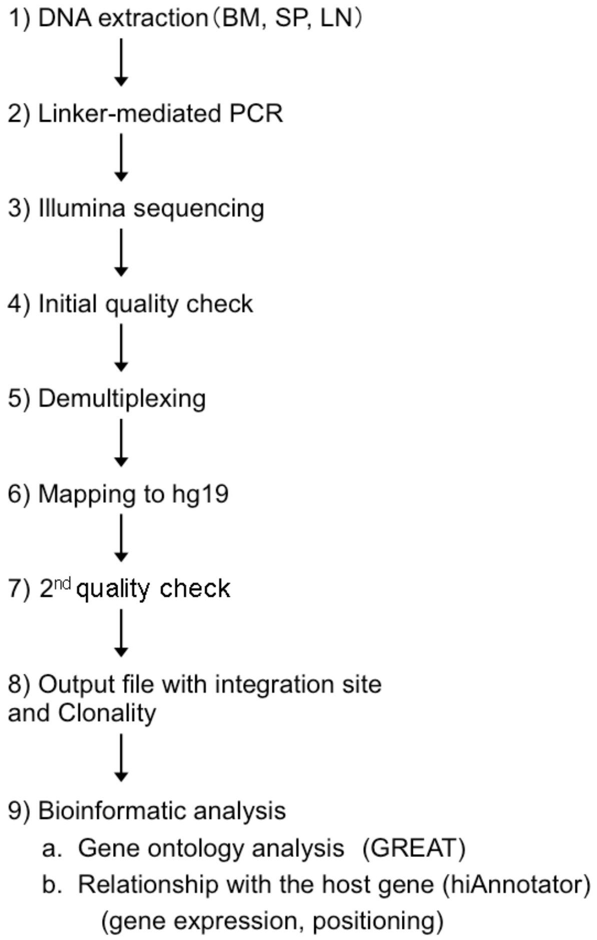

Figure S1. Experimental flow of the clonality analysis of HIV-1-infected cells using the integration site data.

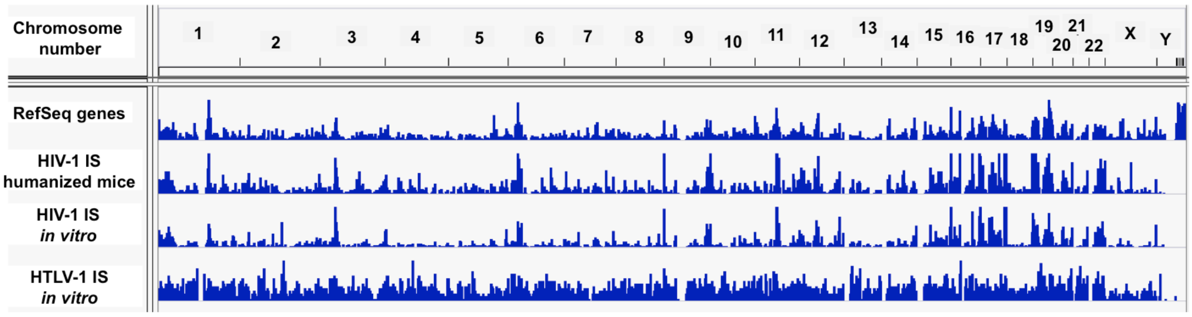

Figure S2. Global view of the distribution of HIV-1 or HTLV-1 integration sites in the host human genome. The distributions of integration sites of HIV-1 *in vitro* or in humanized mice, and HTLV-1 *in vitro* are visualized by Integrative Genomics Viewer. We use here unique IS data of each data set.

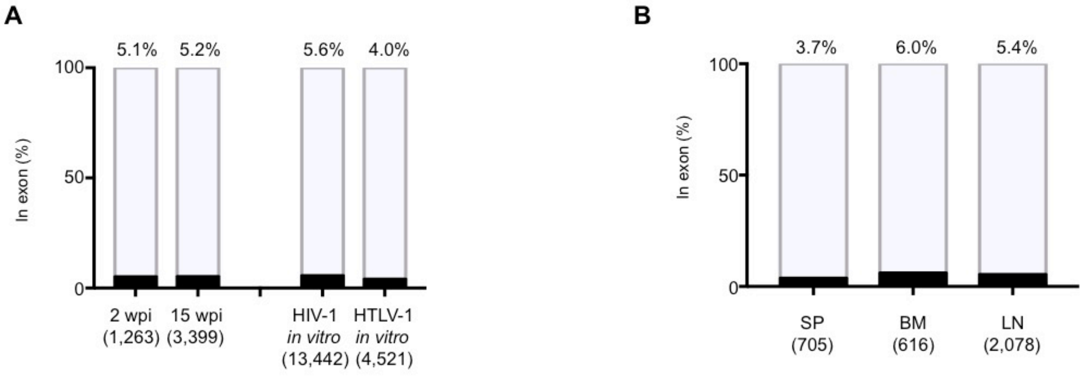

Figure S3. Frequencies of HIV-1 integration into exons of the host genes at different time points (A) or in different tissues (B) of humanized mice. The data obtained from cells infected with HIV-1 or HTLV-1 *in vitro* is also shown as controls.

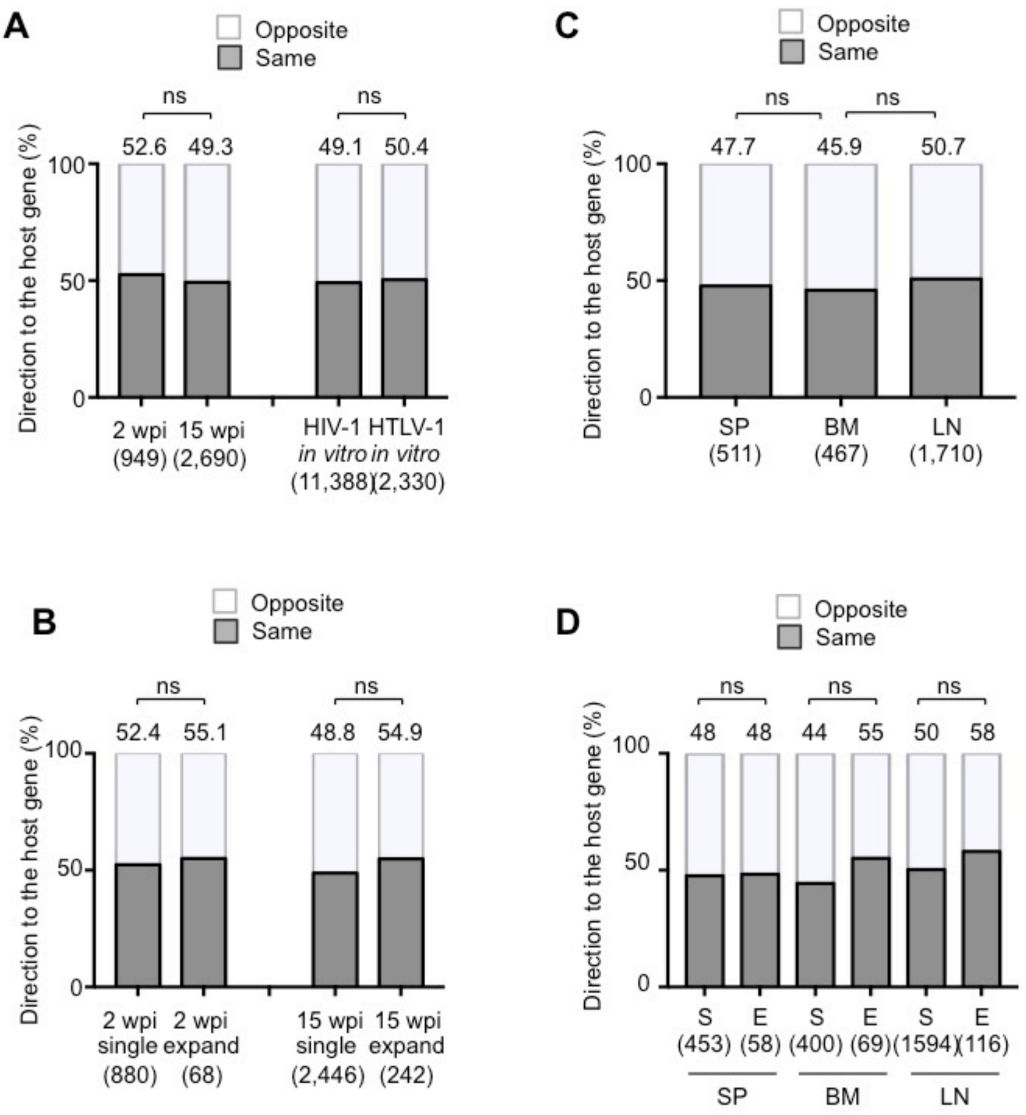

Figure S4. Orientation of the HIV-1 provirus relative to the host genes. (A) Comparison of directionality of HIV-1 provirus in relation to the host gene is shown separately for 2 wpi and 15 wpi mice or *in vitro* data. (B-C) Comparison between expanded and singleton clones is shown for 2 wpi or 15 wpi mice (B) and different tissues (C). (D) Comparison of the provirus orientation between expanded and singleton clones in different tissue. Numbers above the graph indicate the percentage. The number of infected cells or clones in each sample is shown under the graph.

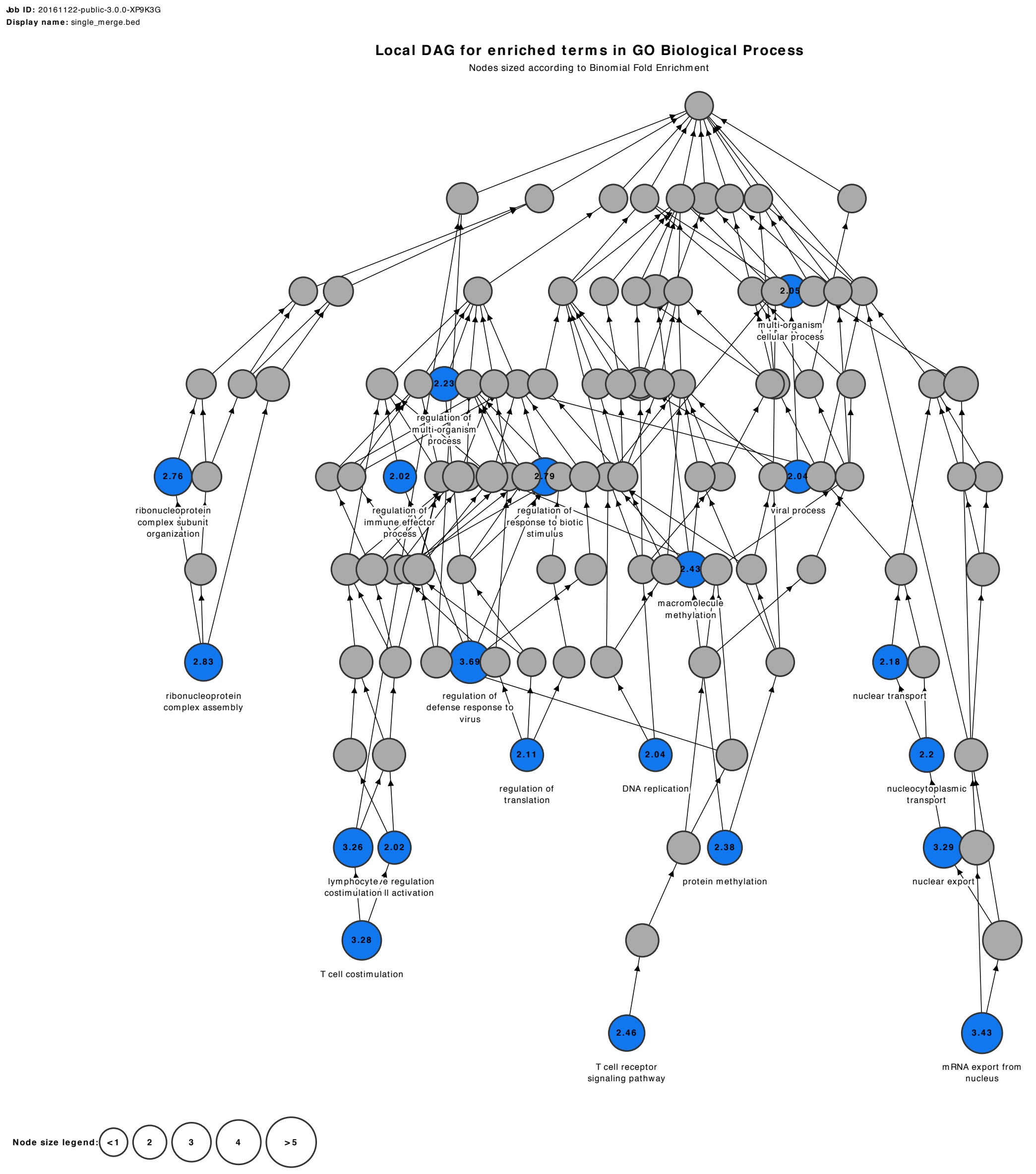

Figure S5. Local directed acyclic graph (DAG) for enriched terms in biological process of gene ontology (GO) in HIV-1 IS of singleton clones.

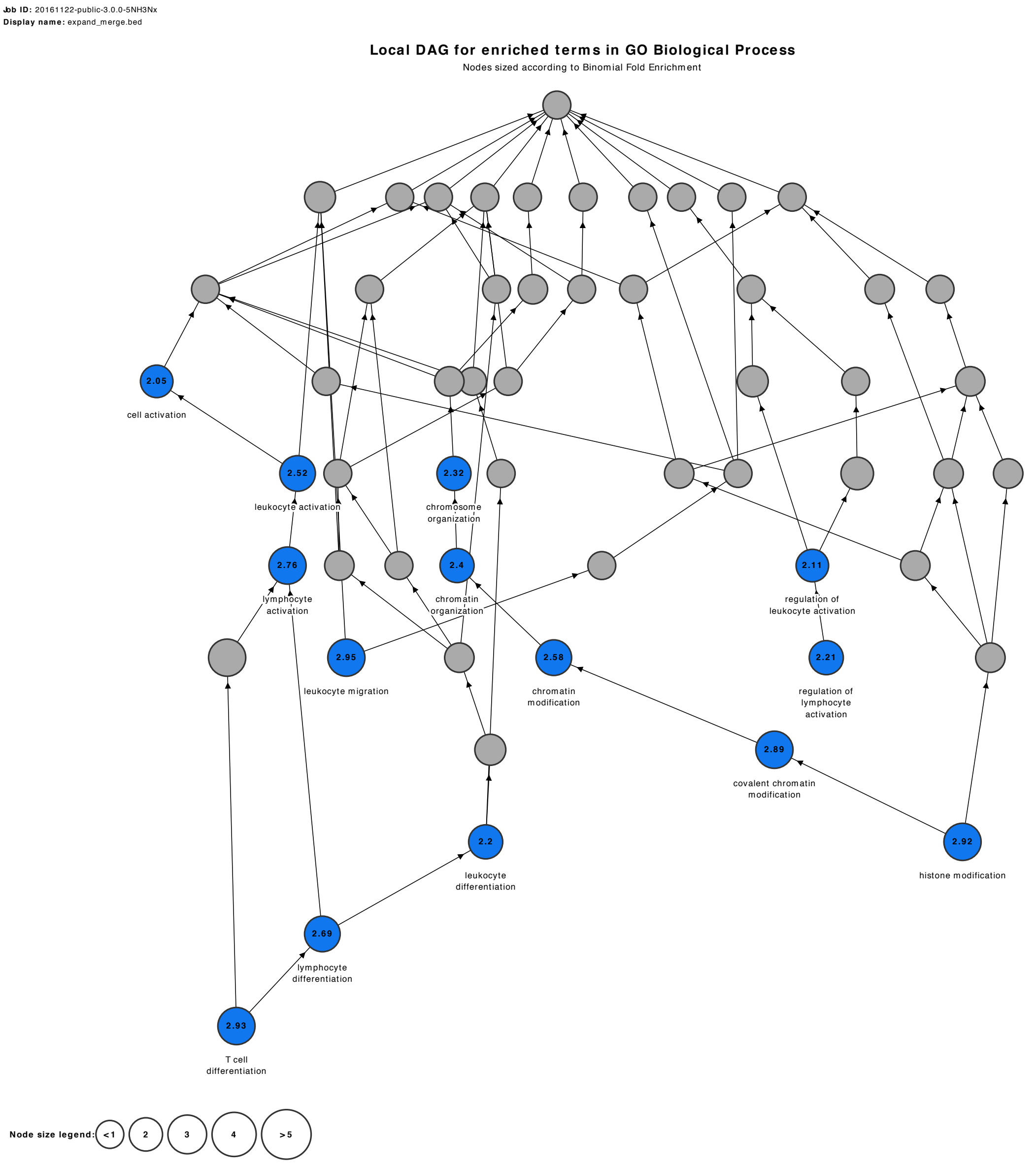

Figure S6. Local DAG for enriched terms in biological process of GO in HIV-1 IS of expanded clones.

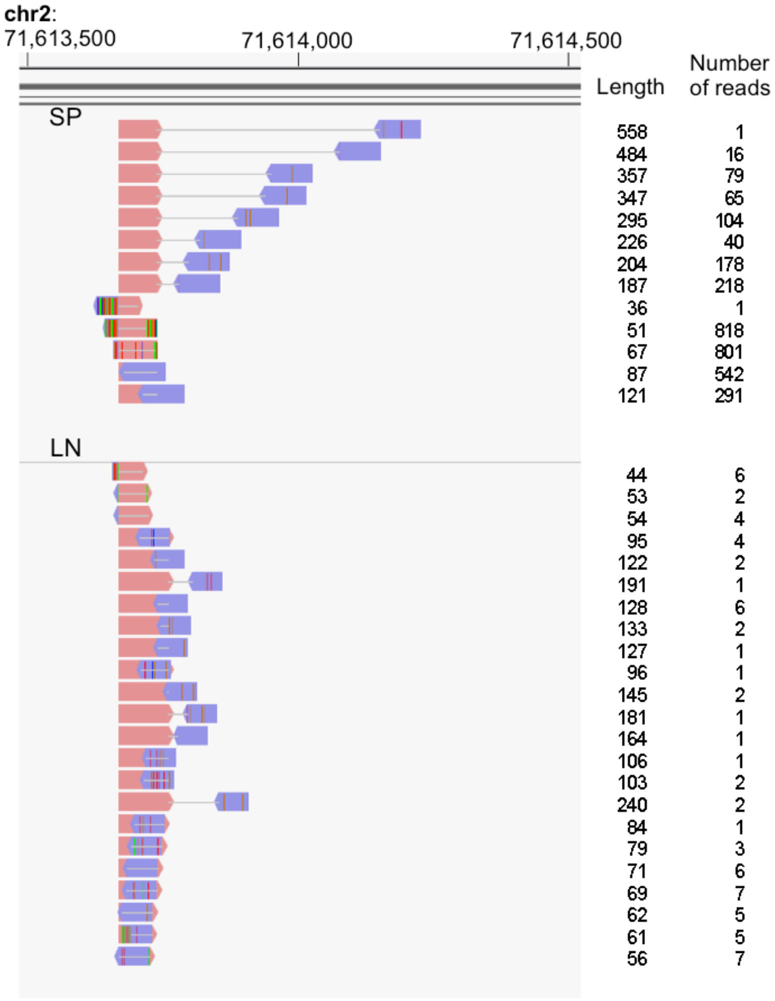

Figure S7. Another clone detected in both SP and LN Is with IS within or near the gene.

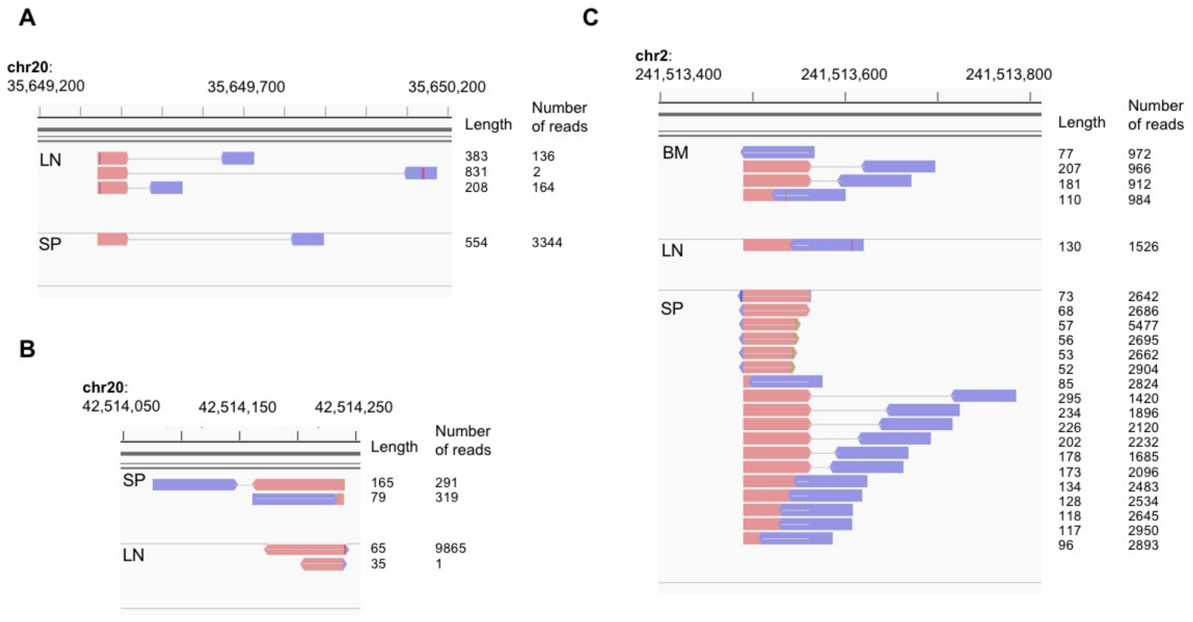

Figure S8. Visualization of integration site data of HIV-1-infefted clones disseminated into multiple organs in mice no.4 (A and B) or no.6 (C).

S1 Table. List of the mice and their tissues analyzed in this study.

Group	Mouse-ID-tissue	Donor ID	CD4 count*	Plasma VL**	PVL (%)	Number of Cleaned Reads	UIS***	% of expanded cells
2 wpi	Mouse-1-SP	1-8	0.29	4.9	1.0	718,700	272	34.7
	Mouse-1-BM				0.1	259,820	34	0.0
	Mouse-2-SP	6-2	0.40	6.2	6.8	778,674	391	13.5
	Mouse-2-BM				14.9	605,601	95	34.7
	Mouse-3-SP	6-1	0.60	6.1	5.6	549,591	291	7.6
	Mouse-3-BM				8.4	601,932	252	10.0
15 wpi	Mouse-4-SP	1-6	0.03	5.0	2.4	613,428	68	32.2
	Mouse-4-BM				2.1	312,433	88	40.7
	Mouse-4-LN				9.1	705,630	778	23.3
	Mouse-5-SP	1-7	0.04	6.5	26.9	1,381,904	385	20.0
	Mouse-5-BM				29.3	706,559	137	10.9
	Mouse-5-LN				23.8	1,745,475	1,119	10.6
	Mouse-6-SP	4-7	0.02	5.6	13.0	904,447	252	38.7
	Mouse-6-BM				7.1	786,860	391	53.3
	Mouse-6-LN				6.4	722,445	181	27.2

* Fold change from initial to end point value in peripheral blood, ** plasma viral load at the end point (log10/mL), UIS; unique integration site.

S2 Table. List of genes associated with HIV-1 integration sites of expanded clones in 2 wpi mice

Gene	# clone	# copy	Description
**ANKRD11**	6	6	Ankyrin Repeat Domain 11
**FASN**	6	9	Fatty Acid Synthase
**MAATS1**	6	6	MYCBP Associated And Testis Expressed 1
**MAPK8IP3**	6	8	Mitogen-Activated Protein Kinase 8 Interacting Protein 3
**NR1I2**	6	6	Nuclear Receptor Subfamily 1 Group I Member 2
**SLC16A3**	6	9	Solute Carrier Family 16 Member 3
**ZNF778**	6	6	Zinc Finger Protein 778
**MANBA**	5	5	Mannosidase Beta
**NFKB1**	5	5	Nuclear Factor Kappa B Subunit 1
**NPLOC4**	5	5	NPL4 Homolog, Ubiquitin Recognition Factor
**PRKCQ**	5	5	Protein Kinase C Theta
**ADRBK1**	4	5	Adrenergic, Beta, Receptor Kinase 1
**CARD8**	4	4	Caspase Recruitment Domain Family Member 8
**CSPG5**	4	5	Chondroitin Sulfate Proteoglycan 5
**CYTH1**	4	4	Cytohesin 1
**DNAH17**	4	4	Dynein Axonemal Heavy Chain 17
**EPB41L2**	4	4	Erythrocyte Membrane Protein Band 4.1 Like 2
**FANCA**	4	5	Fanconi Anemia Complementation Group A
**FKBP5**	4	4	FK506 Binding Protein 5
**GAL**	4	5	Galanin And GMAP Prepropeptide
**GCFC2**	4	4	GC-Rich Sequence DNA-Binding Factor 2
**HN1L**	4	4	Hematological And Neurological Expressed 1-Like
**HORMAD2**	4	4	HORMA Domain Containing 2
**KDM2A**	4	5	Lysine Demethylase 2A
**LRRTM4**	4	4	Leucine Rich Repeat Transmembrane Neuronal 4
**MC1R**	4	6	Melanocortin 1 Receptor
**NPEPPS**	4	4	Aminopeptidase Puromycin Sensitive
**PFKFB3**	4	4	6-Phosphofructo-2-Kinase/Fructose-2,6-Biphosphatase 3
**PPP6R3**	4	5	Protein Phosphatase 6 Regulatory Subunit 3
**PTPN11**	4	4	Protein Tyrosine Phosphatase, Non-Receptor Type 11
**RPH3A**	4	4	Rabphilin 3A
**SCAF11**	4	4	SR-Related CTD Associated Factor 11
**SLC38A1**	4	4	Solute Carrier Family 38 Member 1
**SMARCC1**	4	5	SWI/SNF Related, Matrix Associated, Actin Dependent Regulator Of Chromatin Subfamily C Member 1

clone; the number of infected clones with IS within or near the gene. # copy; the number of infected cells with IS within or near the gene.

S3 Table List of genes associated with HIV-1 integration sites of expanded clones in 15 wpi mice

Gene	# clone	# copy	Description
**DNMT1**	18	19	DNA (Cytosine-5-)-Methyltransferase 1
**EIF3G**	17	17	Eukaryotic Translation Initiation Factor 3 Subunit G
**GPR174**	17	51	G Protein-Coupled Receptor 174
**HSF1**	14	16	Heat Shock Transcription Factor 1
**P2RY10**	14	48	Purinergic Receptor P2Y10
**NFAT5**	11	11	Nuclear Factor Of Activated T-Cells 5, Tonicity-Responsive
**NPLOC4**	11	11	NPL4 Homolog, Ubiquitin Recognition Factor
**DGAT1**	10	12	Diacylglycerol O-Acyltransferase 1
**FCHSD2**	10	10	FCH And Double SH3 Domains 2
**GCFC2**	10	10	GC-Rich Sequence DNA-Binding Factor 2
**KLC2**	10	10	Kinesin Light Chain 2
**MANBA**	10	10	Mannosidase Beta
**PACS1**	10	10	Phosphofurin Acidic Cluster Sorting Protein 1
**TUBD1**	10	27	Tubulin Delta 1
**VMP1**	10	27	Vacuole Membrane Protein 1
**ATG16L2**	9	9	Autophagy Related 16 Like 2
**CARD8**	9	10	Caspase Recruitment Domain Family Member 8
**GLCCI1**	9	10	Glucocorticoid Induced 1
**MAATS1**	9	9	MYCBP Associated And Testis Expressed 1
**NFKB1**	9	9	Nuclear Factor Kappa B Subunit 1
**NQO1**	9	9	NAD(P)H Quinone Dehydrogenase 1
**NR1I2**	9	9	Nuclear Receptor Subfamily 1 Group I Member 2
**RNF19A**	9	11	Ring Finger Protein 19A, RBR E3 Ubiquitin Protein Ligase
**ASH1L**	8	8	Ash1 (Absent, Small, Or Homeotic)-Like (Drosophila)
**CCDC91**	8	8	Coiled-Coil Domain Containing 91
**CHMP6**	8	8	Charged Multivesicular Body Protein 6
**FANCA**	8	9	Fanconi Anemia Complementation Group A
**ITGAL**	8	10	Integrin Subunit Alpha L
**KCNA4**	8	10	Potassium Voltage-Gated Channel Subfamily A Member 4
**LRRTM4**	8	8	Leucine Rich Repeat Transmembrane Neuronal 4
**PPP6R2**	8	9	Protein Phosphatase 6 Regulatory Subunit 2
**RAD51B**	8	49	RAD51 Paralog B
**RPTOR**	8	8	Regulatory Associated Protein Of MTOR Complex 1
**SBF1**	8	9	SET Binding Factor 1
**TOX**	8	9	Thymocyte Selection Associated High Mobility Group Box

clone; the number of infected clones with IS within or near the gene. # copy; the number of infected cells with IS within or near the gene.

S4 Table. Sequences of oligonucleotides used in the linker-mediated PCR (A) and high-throughput sequencing (B).

(A) linker-mediated PCR

Linkers	Sequences (5’-3’)
Long linker:	TCATATAATGGGACGATCACAAGCAGAAGACGGCATACGAGATNNNNNNNN CGGTCTCGGCATTC CTGCTGAACCGCTCTTCCGATCT
Short linker:	p-GATCGGAAGAGCGAAAAAAAAAAAAA

Primers		Sequences (5’-3’)
1st PCR		
	B3:	GCTTGCCTTGAGTGCTTCAAGTAGTGTG
	B4:	TCATGATCAATGGGACGATCA
2nd PCR		
	P5B5:	AATGATACGGCGACCACCGAGATCTACACGTGCCCGTCTGTTGTGTGACTCTGG
	P7:	CAAGCAGAAGACGGCATACGAGAT

(B) High-throughput sequencing

Primers	Sequences (5’-3’)
Read1:	ATCCCTCAGACCCTTTTAGTCAGTGTGGAAAATCTC
Read2:	CGGTCTCGGCATTCCTGCTGAACCGCTCTTCCGATCT
Read3:	GATCGGAAGAGCGGTTCAGCAGGAATGCCGAGACCG

S5 Table. Sequences of PCR primers for Proviral load measurement

Primers	Sequences (5'-3')
Gag-F	GGTGCGAGAGCGTCAGTATTAAG
Gag-R	AGCTCCCTGCTTGCCCATA
Alb-F	TGCATGAGAAAACGCCAGTAA
Alb-R	ATGGTCGCCTGTTCACCAA
2LTR-F	AACTAGGGAACCCACTGCTTAAG
2LTR-R	TCCACAGATCAAGGATATCTTGTC
